# Application value of tissue tuberculosis antigen combined with Xpert MTB/RIF detection in differential diagnoses of intestinal tuberculosis and Crohn’s disease

**DOI:** 10.1186/s12879-021-06210-8

**Published:** 2021-05-28

**Authors:** Baoying Fei, Lin Zhou, Yu Zhang, Linhe Luo, Yuanyuan Chen

**Affiliations:** 1 Department of Gastroenterology, Tongde Hospital of Zhejiang Province, 234 Gucui Road, Hangzhou, 310012 Zhejiang China; 2 Departments of Minimally Invasive Surgery, Tuberculous Experimental Center, Hangzhou Red Cross Hospital, Hangzhou, 310003 Zhejiang China; 3 Department of Gastroenterology, Zhejiang Province People’s Hospital, Hangzhou, 310014 Zhejiang China; 4 Tuberculosis Diagnosis and Treatment Center, Hangzhou Red Cross Hospital, Hangzhou, 310003 Zhejiang China

**Keywords:** Antigen, Crohn’s disease, Diagnosis, Intestinal tuberculosis, Xpert MTB/RIF

## Abstract

**Background:**

The purpose of this study was to examine the value of Xpert MTB/RIF assay and detection of additional *Mycobacterium tuberculosis* complex (MTBC) species antigens from intestinal tissue samples in differentiating intestinal tuberculosis (ITB) from Crohn’s disease (CD).

**Methods:**

Several clinical specimens of intestinal tissue obtained by either endoscopic biopsy or surgical excision were used for mycobacteriologic solid cultures,Xpert MTB/RIF assays, immunohistochemistry, and histological examinations. Four antigens (38KDa, ESAT-6, MPT64, and Ag85 complex) of MTBC in the intestinal tissue were detected by immunohistochemical analysis.

**Results:**

The study included 42 patients with ITB and 46 with CD. Perianal lesions and longitudinal ulcers were more common in patients with CD, while caseating granuloma and annular ulcers were more common in patients with ITB. The positive rate of MTBC detected by Xpert MTB/RIF in intestinal tissues of patients with ITB was 33.33%, which was significantly higher than that in patients with CD and that detected using acid-fast staining smears. It was also higher than that detected by tissue MTBC culture, but the difference was not statistically significant. The positive MPT64 expression rate in patients with ITB was 40.48%, which was significantly higher than that observed in patients with CD. The sensitivity of parallelly combined detection of tuberculosis protein MPT64 and Xpert MTB/RIF in diagnosing ITB was 50.0%.

**Conclusions:**

The detection of Xpert MTB/RIF in intestinal tissue is a rapid and useful method for establishing an early diagnosis of ITB. The detection of MTBC using Xpert MTB/RIF and MPT64 antigen in intestinal tissues has a definitive value in the differential diagnosis ofITB and CD. The combination of these two methods can improve the detection sensitivity.

## Background

ITB and CD are chronic intestinal granulomatous diseases that share many similarities in clinical manifestations, imaging, endoscopy and intestinal histopathology. Over recent years, the incidence of tuberculosis and ITB has significantly increased following the population migration, drug-resistant bacterial infection, and spread of AIDS [1]. The incidence of CD also continues to increase in China [2]. Several studies addressed the clinical, pathological and molecular biological detections of the two diseases. However, the differential diagnosis of ITB and CD still represents a challenging clinical problem, especially in the developing countries with a high incidence of tuberculosis where the misdiagnosis and missed diagnosis may lead to serious adverse consequences [3–5].

Xpert MTB/RIF is a new technology recently approved by the World Health Organization (WHO) [6]. Xpert MTB/RIF is an in vitro diagnostic technique based on semi-nested multiplex real-time fluorescent quantitative PCR using Gene Xpert full-automatic closed nucleic acid detection system. Primers and probes are designed to target the core sequence of rifampicin-resistant determinant regions which are characteristic of MTBC species. It can be used to not only diagnose MTBC infection, but also determine rifampicin resistance (mutation in rpoB sequence). The whole detection process can be completed in 2 h. Since its first applications, this technology has been gradually recognized and approved for its diagnostic value in tuberculosis detection, showing high accuracy, sensitivity of 89% and specificity of 98% in diagnosing adult pulmonary tuberculosis [7]. Previous studies investigated the diagnostic value of Xpert MTB/RIF in extrapulmonary tuberculosis [8–10]. Nevertheless, studies on the application of Xpert MTB/RIF in diagnosing of ITB are still very rare.

MTBC species secrete a variety of antigens with potential diagnostic significance [11–13]. 38KDa protein is a membrane protein, closely related to the transport of phosphate. It is an early secretory protein encoded by the *Psts* gene that can stimulate T cells and B cells to develop corresponding immune response. ESAT-6 is an early secretory protein that exists only in MTBC and a few pathogenic mycobacteria, but not in non-pathogenic mycobacteria and BCG. MPT64 exists in MTBC, and most BCG strains cannot secrete this protein. The Ag85 complex is an acid transferase in Mycobacterium, where each strain of Mycobacterium can secrete Ag85, which has an important role in the late stage of cell wall synthesis of MTBC.

In this study, the Xpert MTB/RIF detection system was used to detect MTBC. The immunohistochemical method was used to detect several tuberculosis antigens in the intestinal tissue samples of patients with ITB and CD so as to clarify the role of Xpert MTB/RIF detection and tuberculosis antigen detection in the differential diagnosis of ITB and CD. Additionally, the clinical, endoscopic and pathological features of CD and ITB were also compared.

## Methods

### Patients

Patients clinically suspected of having CD and ITB between January 2016 and March 2018 were initially included in this study. Patients who received antituberculosis treatment within 3 months before the collection of specimens were excluded. The clinical, endoscopic, radiological and biopathological data of the participants were collected, and the patients were followed up after treatment. Several specimens of intestinal tissue from endoscopic biopsy or surgical excision were collected. Four endoscopic biopsy samples were used to make into paraffin-embedded specimen, and another four to six endoscopic biopsy samples were used to detect MTBC using culture and Xpert MTB/RIF.

The written informed consent was obtained from all the patients before the clinical trials started and the study was approved by the ethics committee of Tongde Hospital of Zhejiang Province.

### Diagnostic criteria

The ITB diagnosis was established if at least one of the following criteria was met [14]: 1) histological evidence of a caseating granuloma, 2) histological demonstration of acid-fast bacilli (AFB), 3) intestinal granulomatous inflammation accompanied by histologically or microbiologically confirmed extraintestinal TB, and 4) a positive MTBC culture. The CD diagnosis was based on the European Crohn’s and Colitis Organization guidelines, using a combination of clinical, endoscopic, radiological, and histological features [15].

In patients for whom the differentiation between ITB and CD was uncertain, anti-TB therapy (ATT) was attempted for 2–3 months, and the final diagnosis was made based on the clinical and endoscopic response to the anti-TB therapy [16, 17]. The clinical response was determined by the loss of subjective symptoms. The endoscopic response was determined by the disappearance of ulcerations.

### Xpert MTB/RIF detection of intestinal tissue specimens

The intestinal tissue specimens were decontaminated with 1% N-acetyl-L-cysteine NaOH (NALC-NaOH) and then centrifuged. The tissue was mechanically homogenized and suspended in 1 mL of sterile saline solution. About 1 mL of the specimen was taken, and two times volume of sample treatment solution was added. The solution was mixed by vortex oscillation for 10 s, kept at room temperature for 10 min, mixed again by vortex oscillation for 10 s, and then kept at room temperature for 5 min. The mixture of 2 mL was added to an Xpert reagent kit, and the kit was put into a Gene Xpert instrument for detection. After the reaction, the test results were directly observed under the window of a detection system.

### Acid-fast staining and MTBC culture of intestinal tissues

Fresh intestinal tissue specimens were decontaminated with 1% NALC-NaOH and then centrifuged. The tissue was mechanically homogenized and suspended in sterile saline solution. The treated samples were examined by acid-fast staining smear,and MTBC culture with Lowenstein-Jensen (LJ) medium. The specific procedures were carried out in accordance with "The Laboratory Science Procedure of Diagnosis Bacteriology in Tuberculosis".

### Immunohistochemical detection of several tuberculosis antigens in intestinal tissues

Four antigens of MTBC (38KDa protein, ESAT-6 protein, MPT64,and Ag85 complex) were detected. Paraffin-embedded slices (4 μm thick) were taken. Dewaxing, thermal repair and blocking were sequentially performed. The primary antibody was incubated overnight, and the secondary antibody was incubated for 1 h. The slices were washed with PBS, stained with DAB for 5 min, and sealed. The procedure strictly followed the instructions on the kit.

### Statistical analysis

SPSS20.0 software was used for statistical analyses. Measurement data were expressed as mean ± standard deviation and t test was used for comparison between groups. Countable data were expressed in rates, and the *χ*^2^ test or Fisher’s exact probability method was used for comparison between groups. A *P* < 0.05 indicated a statistically significant difference.

## Results

### General information

Histological specimens were initially collected from 110 patients; of these, 22 patients were excluded for diagnosing non-ITB or CD (17 patients with nonspecific small bowel ulcers,3 with ulcerative colitis, and 2 with intestinal Behcet’s disease). Finally, 42 patients with ITB (14 were surgical specimens) and 46 with CD (3 were surgical specimens) were included in the experimental analyses. Symptomatic improvement and endoscopic healing were noted in 22 of the 30 patients started on ATT for probable ITB.

Further,20 male (47.6%) and 22 female (52.4%) patients had ITB. The age of onset was 18–59 years and the mean age was 30.5 ± 11.8 years. Also, 27 male (58.7%) and 19 female (41.3%) patients had CD. The age of onset was 17–61 years and the mean age was 31.5 ± 12.2 years. No significant difference was found in sex and age between the two groups.

### Clinical, endoscopic, and histological features

Clinical,endoscopic, and histological features are shown in Table 1. The most common symptoms of both CD and ITB were abdominal pain and diarrhea. Abdominal pain was found in 41 patients (89.1%) with CD and 33 patients (78.6%) with ITB. Diarrhea was found in 37 patients (80.4%) with CD and 29 patients (69.0%) with ITB. No significant difference was noted in abdominal pain and diarrhea among the groups. Moreover, 10 (21.7%) patients with CD had perianal lesions, including 6 withanal fistula, 3 with perianal abscess and 1 with anal fissure; 1 patient with ITB had a perianal lesion (anal fissure). The observed difference was significant (*P* < 0.01). A longitudinal ulcer was found in 17 patients with CD (36.9%), which was significantly higher than that in patients with ITB (6 patients, 14.3%). Annular ulcer was found in 25 patients with ITB (59.5%) which was significantly higher than that in patients with CD (7 patients, 15.2%). Among patients with CD, 11 (23.9%) had aphthous ulcer, 10 (21.7%) had paving-stone like changes in ulcers and 11 (23.9%) had pseudopolyps, which were significantly different from those found in patients with ITB (4 patients, 9.5%; 5 patients, 11.9%; 14 patients, 33.3%); the observed differences were not statistically significant. Caseating granuloma was found in three cases of ITB endoscopic biopsy specimens and seven cases of ITB surgical specimens, which had definitive diagnostic significance. Non- Caseating granulomas were found in 17 patients with ITB (40.5%) and 12 patients with CD (26.1%), but with no significant difference between the groups. The difference in lymphocyte aggregation at the bottom of the lamina propria between the two groups was also non-significant.

**Table 1:** Clinical, endoscopic, and histological features in the differentiation of ITB and CD

	ITB (*n* = 42)	CD (*n* = 46)	***P***-value
**Clinical presentation**			
Age, years (mean ± sd)	30.5 ± 11.8	31.5 ± 12.2	0.83
Sex (male:female)	20:22	27:19	0.10
Abdominal pain	33 (78.6%)	41 (89.1%)	0.10
Diarrhea	29 (69.0%)	37 (80.4%)	0.09
Perianal disease	1 (0.03%)	10 (21.7%)	0.01
**Endoscopic features**			
Longitudinal ulcer	6 (14.3%)	17 (36.9%)	0.01
Annular ulcer	25 (59.5%)	7 (15.2%)	0.01
Aphthous ulcers	4 (9.5%)	11 (23.9%)	0.05
Cobblestone appearance	5 (11.9%)	10 (21.7%)	0.11
Pseudopolyps	14 (33.3%)	11 (23.9%)	0.12
**Histological features**			
Caseous granuloma	10 (23.8%)	0 (0.00%)	0.01
Non-caseous granulomas	17 (40.5%)	12 (26.1%)	0.07
Lymphocyte aggregation	19 (45.2%)	17 (37.0%)	0.13

### Detection and comparison of MTBC in intestinal tissues of patients with ITB and CD

The positive rate of MTBC detected by Xpert MTB/RIF in intestinal tissues of patients with ITB was 33.33%, which was significantly higher than that of patients with CD (*P* < 0.01). The positive rate detected by Xpert MTB/RIF in 28 biopsy specimens of patients with ITB and 14 surgical specimens was 14.28% (4/28) and 71.43% (10/14) respectively. None of them showed resistance to RIF. The positive rate of MTBC detected by Xpert MTB/RIF in intestinal tissue specimens of patients with ITB (33.33%) was higher than that detected by tissue MTBC culture (21.43%), but the difference was not statistically significant. The positive rate detected by tissue MTBC culture in biopsy specimens of 28 patients with ITB and 14 surgical specimens was 7.14% (2/28) and 50% (7/14), respectively. The positive rate of MTBC detected by Xpert MTB/RIF in intestinal tissue specimens of patients with ITB (33.33%) was significantly higher than that detected using acid-fast staining smear (11.9%). The results are shown in Table 2.

**Table 2:** Detection and comparison of MTBC in intestinal tissues of patients with ITB and CD

	ITB (*n* = 42)	CD (*n* = 46)
Xpert MTB/RIF(+)	14 (33.3%)	0
MTBC Culture(+)	9 (21.4%)	0
Acid-fast Staining Smear(+)	5 (11.9%)	1 (2.17%)

### Clinical evaluation of the detection of *MTBC* in intestinal tissues by Xpert MTB/RIF

The specificity of all three methods for detecting MTBC in intestinal tissues was very high. The specificity and positive predictive value of Xpert MTB/RIF for detecting MTBC in intestinal tissues were both 100%. The negative predictive value of Xpert MTB/RIF for detecting MTBC in intestinal tissues (62.2%) was higher than that of tissue MTBC culture (58.2%) and acid-fast staining (54.9%), with no significant difference. The results are shown in Table 3**.**

**Table 3:** Clinical evaluation of the detection of MTBC in intestinal tissues

	sensitivity (95%CI)	specificity (95%CI)	PPV (95%CI)	NPV (95%CI)
Xpert TB/RIF	33.3% (20.0–49.6)	100% (90.4–100)	100% (73.2–100)	62.2% (50.1–73.0)
MTBC culture	21.4% (10.8–37.2)	100% (90.4–100)	100% (62.9–100)	58.2% (46.6–69.1)
Acid-fast Staining Smear	11.9% (4.5–26.4)	97.8% (87.0–99.9)	83.3% (36.5–99.1)	54.9% (43.5–65.8)

### Expression of intestinal TB proteins in patients with ITB and CD

**Fig. 1:**
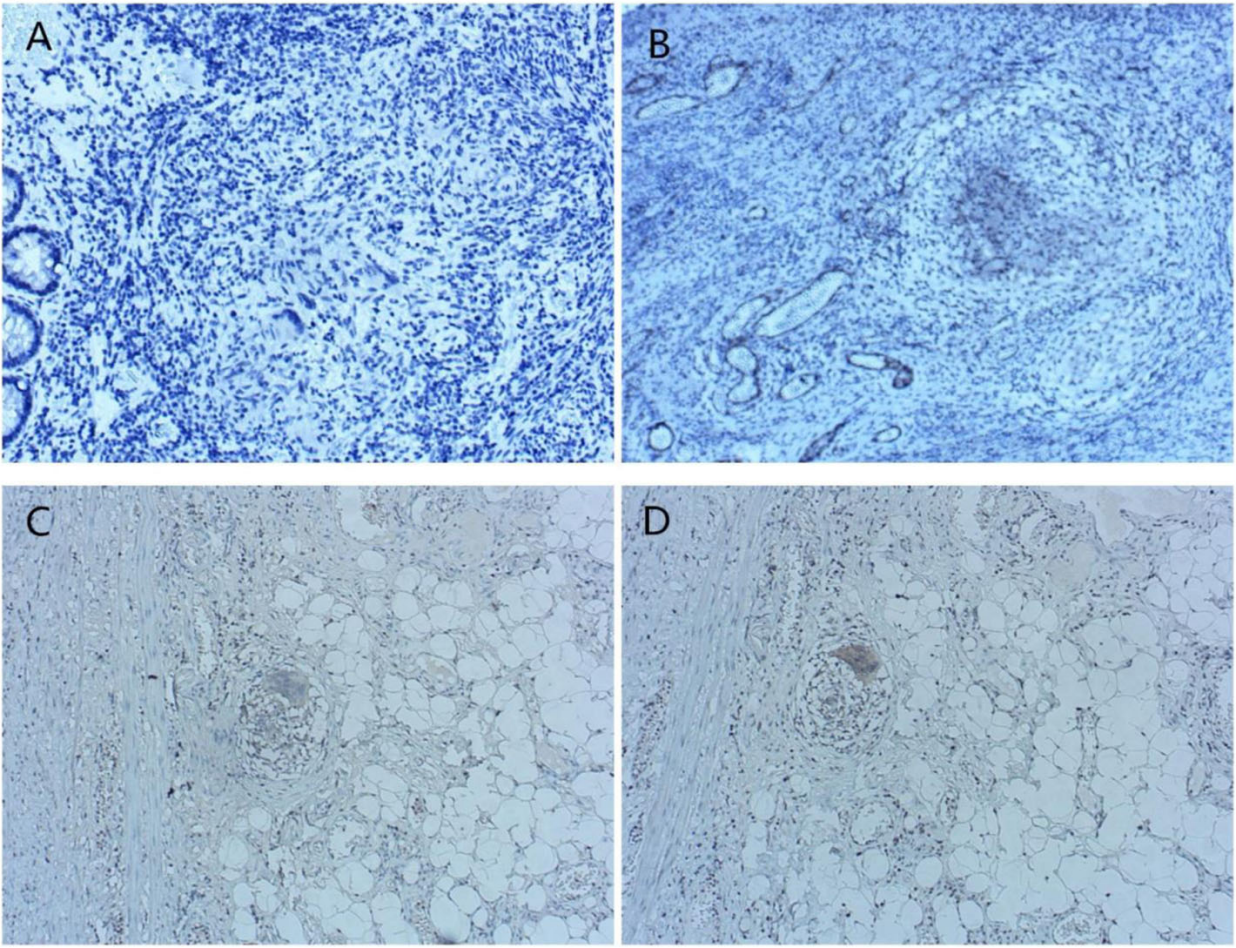
four tuberculosis proteins immunohistochemical view of the colonic specimen of ITB or CD patients (× 200). **A**: 38KDa; **B**: MPT64; **C**: Ag85B; **D**: ESAT-6

38KDa, ESAT-6, MPT64, and Ag85 complex were expressed in the granular cytoplasm of intestinal granuloma cells in ITB and CD (Fig.1). The positive rate of MPT64 expression was 40.48% in patients with ITB and 19.56% in patients with CD, and the difference was statistically significant (*χ*^2^ = 4.61, *P* < 0.05). However, no statistically significant difference was found in the expression of the other three tuberculosis proteins between the two diseases (Table 4).

**Table 4:** Expression of intestinal TB proteins in patients with ITB and CD

	ITB (*n* = 42)	CD (*n* = 46)	***P***-value
38KDa(+)	4 (9.52%)	9 (19.56%)	0.1
MPT64 (+)	17 (40.48%)	9 (19.56%)	0.02
Ag85B (+)	10 (23.81%)	11 (23.91%)	0.2
ESAT-6 (+)	11 (26.19%)	15 (32.61%)	0.15

### Detection of MTBC using intestinal TB proteins combined with Xpert MTB/RIF

The sensitivity and specificity of parallelly combined detection of tuberculosis protein MPT64 and Xpert MTB/RIF in diagnosing ITB were 50.0 and 80.44%, respectively (Table 5). The sensitivity of combined detection improved compared with the sensitivity of single detection, but the specificity declined.

**Table 5:** Detection of MTBC using intestinal TB protein combined with Xpert MTB/RIF

	ITB (*n* = 42)	CD (*n* = 46)
Xpert MTB/RIF(+)or MPT64(+)	21 (50.0%)	9 (19.56%)
Xpert MTB/RIF(−)and MPT64(−)	21 (50.0%)	37 (80.44%)

## Discussion

CD and ITB share many similarities in clinical manifestations and signs, which makes their differentiation difficult [18]. Some studies have proposed certain criteria for identifying CD and ITB, but these criteria have been shown to have many limitations [19–21]. In this study, common symptoms of CD and ITB, such as abdominal pain, diarrhea and ascites, had no significant differences between the two groups. The number of perianal lesions was significantly higher in patients with CD than in patients with ITB, suggesting that perianal lesions had certain value for the differentiation of the two diseases. Perianal lesions are often the first symptom of CD and closely related to disease activity. Therefore, clinicians should pay close attention to them. The present study showed that the incidence of longitudinal ulcer and stenosis was significantly higher in patients with CD than in patients with ITB. Also, the incidence of annular ulcer was significantly higher in patients with ITB than in patients with CD. These data were consistent with previous findings [22, 23] and suggested that the longitudinal ulcer and stenosis were the characteristic manifestations of CD and the annular ulcer was the characteristic manifestation of ITB. The pathological changes in CD and ITB usually occur in the submucosa of the intestinal wall. Because of mucosal swelling, the endoscopic biopsy tissues tend to be small and the sampling is superficial, resulting in low positive rates. In the present study, only three cases of caseating granuloma were detected in ITB endoscopic biopsy, while the pathological positive rate was much lower than that of surgical specimens, suggesting that the differential diagnostic value of endoscopic biopsy specimens in the two diseases was much lower than that of surgical specimens. Non-caseating granuloma was more common in patients with ITB than in patients with CD, but with no statistically significant difference. Non-caseating granuloma alone is not enough to completely exclude ITB and should not be considered as a CD-specific manifestation.

At present, the WHO recommends Xpert MTB/RIF for the rapid diagnosis of pulmonary tuberculosis and some forms of extrapulmonary tuberculosis [24, 25]. Reports on Xpert MTB/RIF detection in intestinal mucosa in China and abroad are few [26, 27]. In the present study, the intestinal tissue specimens from 42 patients with ITB and 46 with CD were examined by Xpert MTB/RIF. The results showed that the positive rate of MTBC detected by Xpert MTB/RIF in intestinal tissue samples of patients with ITB was 33.33%, which was higher than that of patients with CD (0%), and the specificity was 100%. This finding suggested that the detection of Xpert MTB/RIF in intestinal mucosa might have an important role in the differential diagnosis of ITB and CD. In a retrospective study in India,of 37 patients with ITB, Xpert MTB/RIF analysis was performed on colonic biopsy samples from 37 patients with ITB, revealing that the assay had low sensitivity (8.1%) [27]. In this study, the positive rate of MTBC detected by Xpert MTB/RIF was much lower in endoscopic biopsy specimens compared with surgical specimens, which might be because the endoscopic biopsy specimens were superficial and small. MTBC was not evenly distributed in intestinal tissues, and low levels of MTBC extracted from mucosal biopsy specimens might lead to false-negative results of Xpert MTB/RIF in tissues. The new Xpert MTB/RIF Ultra (Xpert Ultra) has been recently developed to boost the sensitivity for the detection of MTBC not only in patients with paucibacillary TB, but also in pediatric patients with TB and those with extrapulmonary TB [28, 29]. However, the increase in sensitivity provided by Xpert Ultra came at the expense of a modest reduction in specificity.

The gold standard for the etiological diagnosis of tuberculosis is *MTBC* culture. However, the growth of M*TBC* usually takes 3–8 weeks, and the positive rate of culture is low, which often causes difficulties in diagnosis. Although the routine acid-fast staining method takes only 2–3 h, this approach has very low sensitivity and specificity of strain identification. It can detect the acid-fast *Mycobacterium*, but it cannot distinguish *MTBC* from other mycobacteria. The sensitivity and accuracy of PCR for pathogen detection provides a useful and new method for diagnosing *MTBC* [30–32]. In this study, the detection of MTBC in the intestinal mucosa by Xpert MTB/RIF was compared with the detection by traditional methods. The results showed that the sensitivity of Xpert MTB/RIF was 33.3%, which was significantly higher than that of acid-fast staining (11.9%) and higher than that of bacillus culture (21.4%). The results indicated that Xpert MTB/RIF had superior sensitivity compared with acid-fast staining in detecting MTBC. It also had superior detection time and higher specificity compared with MTBC culture. Therefore, the Xpert MTB/RIF was better approach for the early diagnosis and treatment of ITB.

MTBC culture with Mycobacterium Growth Indicator Tube (MGIT) system was reported to be positive in 20–42% of colonoscopic biopsy specimens [33, 34]. In the present study,the positive rate of MTBC detected by tissue culture in patients with ITB was 21.43%(7.14% in biopsy specimens and 50% in 14 surgical specimens, LJ medium). The lower yield of MTBC culture in biopsy specimens might be associated with the culture medium and the number of colonoscopic biopsies. The yield of MTBC culture on the LJ medium was lower than that on the MGIT system. However, the false-positive rates for the MGIT system was higher than those for LJ methods [35]. A recent report showed that an additional four (total eight) colonoscopic biopsies improved the yield of TB culture positivity over four biopsies by 11.4–14.3% [34]. Increasing the number of colonoscopic biopsy specimens to eight should be considered for improving AFB culture positivity, although it takes more time.

Increasing attention has been paid to the study of MTBC antigen following the development of immune technology in recent years [13, 36]. At present, the main samples used for MTBC antigen diagnosis are body fluid and bacterial culture medium [37, 38]. Reports on the application of tuberculosis antigen in ITB tissues are very rare. Only two reports on 38KDa antigen with a limited number of ITB cases (≤10 cases) are available. Ihama et al. performed the immunohistochemical detections of 38KDa antigen in intestinal paraffin-embedded specimens of 10 patients with ITB, revealing that 40% of the patients were positive, which in turn suggested that immunohistochemical detection with MTBC monoclonal antibody might be an effective and simple diagnostic method for ITB [39]. Ince and colleagues performed the immunohistochemical detection of 38KDa antigen in 45 tissue specimens (including 8 colon tissue specimens, 8 skin specimens, 5 lung tissue specimens and 24 lymph node specimens). They found that 73% of patients with TB were positive, while only 2 of 28 patients with CD were positive, suggesting that immunohistochemical detection of 38KDa antigen could be used to distinguish ITB from CD [40]. Currently, no reports are available on the expression of pstS1 (38KDa) and East-6 antigens in intestinal tissues. The results of this study showed that the expression of Ag85B, pstS1 (38KDa) and ESAT-6 antigen proteins in ITB and CD had no significant difference. The positive expression rates of Ag85B, pstS1 (38KDa) and ESAT-6 antigen in intestinal tissues were 9.52, 23.8 and 26.2%, respectively. The sensitivity of the immunostaining detection of these three proteins in intestinal tissues was low and could not be improved by their combined detection. Future studies should investigate whether other detection methods such as PCR could improve the sensitivity.

Jørstad et al. used immunohistochemistry to detect the MPT64 antigen in 132 cases of extrapulmonary tuberculosis and non-tuberculosis specimens. MPT64 detection had the best performance in patients with TB lymphadenitis and children with TB, suggesting that MPT64 detection could be used for routine diagnosis under low resource allocation to improve the diagnosis of extrapulmonary tuberculosis, especially for patients with TB lymphadenitis and children with TB [41]. Purohit et al. studied 51 cases of pulmonary tuberculosis and 38 control specimens of non-pulmonary tuberculosis. The results suggested that anti-MPT64 immunostaining detection was a rapid and sensitive method for the early specific diagnosis of *M.* tuberculosis infection [42]. This technique can be easily used in routine pathological laboratories. Currently, no reports exist on the expression of MPT64 antigen in intestinal tissues. In this study, the positive rates of MPT64 antigen in ITB and CD were 40.48 and 19.58%, respectively. The observed difference was statistically significant, suggesting that the MPT64 antigen had definitive value in the differential diagnosis of ITB and CD. Sharma et al. used real-time immuno-PCR (RT-I-PCR) assay for the quantitative detection of a cocktail of mycobacterial MPT64 (Rv1980c) and PstS1 (Rv0934) in patients with TB. The RT-I-PCR assay revealed a high sensitivity of 83.3%, especially for the rapid diagnosis of smear-negative pulmonary TB and paucibacillary extrapulmonary TB [43]. More studies are needed to investigate whether other methods such as PCR may improve the sensitivity of MPT64 antigen detection in intestinal tissues.

## Conclusions

In a word, the detection of Xpert MTB/RIF in intestinal tissue is a rapid and useful method for establishing an early diagnosis of ITB. The detection of Xpert MTB/RIF and MPT64 antigen in intestinal tissues has definitive value in the differential diagnosis of ITB and CD. The combination of these two methods can improve detection sensitivity.

## Data Availability

The datasets used and/or analysed during the current study are available from the corresponding author on reasonable request. Authors have gained informed consent for publication of the dataset from participants at the point of recruitment to the trial.

## Abbreviations

MTBC: *Mycobacterium tuberculosis* cluster
ITB: Intestinal tuberculosis
CD: Crohn’s disease

## References

[1] Glaziou P, Floyd K, Raviglione MC (2018). Global epidemiology of tuberculosis. Semin Respir Crit Care Med.

[2] Cui G, Yuan A (2018). A Systematic Review of Epidemiology and Risk Factors Associated With Chinese Inflammatory Bowel Disease. Front Med (Lausanne).

[3] Kedia S, Das P, Madhusudhan KS, Dattagupta S, Sharma R, Sahni P, Makharia G, Ahuja V (2019). Differentiating Crohn's disease from intestinal tuberculosis. World J Gastroenterol.

[4] Seo H, Lee S, So H, Kim D, Kim SO, Soh JS, Bae JH, Lee SH, Hwang SW, Park SH, Yang DH, Kim KJ, Byeon JS, Myung SJ, Yang SK, Ye BD (2017). Temporal trends in the misdiagnosis rates between Crohn's disease and intestinal tuberculosis. World J Gastroenterol.

[5] Wei JP, Wu XY, Gao SY, Chen QY, Liu T, Liu G (2016). Misdiagnosis and Mistherapy of Crohn's disease as intestinal tuberculosis: case report and literature review. Medicine (Baltimore).

[6] Geneva: World Health Organization (2014). Xpert MTB/RIF implementation manual: Technical and operational ‘How-to’ practical considerations.

[7] Steingart KR, Schiller I, Horne DJ, Pai M, Boehme CC, Dendukuri N. Xpert(R) MTB/RIF assay for pulmonary tuberculosis and rifampicin resistance in adults. Cochrane Database Syst Rev. 2014;(1):CD009593. 10.1002/14651858.CD009593.pub3 PMC4470349 24448973

[8] Mazzola E, Arosio M, Nava A, Fanti D, Gesu G, Farina C (2016). Performance of real-time PCR Xpert ®MTB/RIF in diagnosing extrapulmonary tuberculosis. Infez Med.

[9] Kohli M, Schiller I, Dendukuri N, Yao M, Dheda K, Denkinger CM (2018). Xpert® MTB/RIF assay for extrapulmonary tuberculosis and rifampicin resistance. Cochrane Database Syst Rev.

[10] Prakash AK, Datta B, Tripathy JP, Datta B, Goyal P, Tripathy JP (2018). The clinical utility of cycle of threshold value of GeneXpert MTB/RIF (CBNAAT) and its diagnostic accuracy in pulmonary and extra-pulmonary samples at a tertiary care center in India. Indian J Tuberc.

[11] Gutlapalli R, Sykam A, Tenali SP, Chandran P, Suneetha S, Suneetha LM (2016). Detection of tuberculosis in HIV co-infected individuals: use of multiple ELISA responses to 38kDa, Lipoarabinomannan and ESAT- 6 of M. tuberculosis. J Clin Diagn Res.

[12] Bai L, Chen Y, Bai Y, Liu J, Chen H, Wei Y (2017). Fullerene-doped polyaniline as new redox nanoprobe and catalyst in electrochemical aptasensor for ultrasensitive detection of Mycobacterium tuberculosis MPT64 antigen in human serum. Biomaterials.

[13] Karbalaei Zadeh Babaki M, Soleimanpour S, Rezaee SA (2017). Antigen 85 complex as a powerful *Mycobacterium tuberculosis* immunogene: Biology, immune-pathogenicity, applications in diagnosis, and vaccine design. Microb Pathog.

[14] Lee YJ, Yang SK, Byeon JS, Myung SJ, Chang HS, Hong SS, Kim KJ, Lee G, Jung HY, Hong WS, Kim JH, Min Y, Chang S, Yu C (2006). Analysis of colonoscopic findings in the differential diagnosis between intestinal tuberculosis and Crohn's disease. Endoscopy.

[15] Travis SP, Stange EF, Lémann M, Oresland T, Chowers Y, Forbes A, D'Haens G, Kitis G, Cortot A, Prantera C, Marteau P, Colombel JF, Gionchetti P, Bouhnik Y, Tiret E, Kroesen J, Starlinger M, Mortensen NJ, European Crohn's and Colitis Organisation (2006). European evidence based consensus on the diagnosis and management of Crohn's disease: current management. Gut.

[16] Pratap Mouli V, Munot K, Ananthakrishnan A, Kedia S, Addagalla S, Garg SK, Benjamin J, Singla V, Dhingra R, Tiwari V, Bopanna S, Hutfless S, Makharia G, Ahuja V (2017). Endoscopic and clinical responses to anti-tubercular therapy can differentiate intestinal tuberculosis from Crohn's disease. Aliment Pharmacol Ther.

[17] Sharma V, Mandavdhare HS, Dutta U (2018). Letter: mucosal response in discriminating intestinal tuberculosis from Crohn's disease-when to look for it?. Aliment Pharmacol Ther.

[18] Ma JY, Tong JL, Ran ZH (2016). J intestinal tuberculosis and Crohn's disease: challenging differential diagnosis. Dig Dis.

[19] Limsrivilai J, Shreiner AB, Pongpaibul A, Laohapand C, Boonanuwat R, Pausawasdi N (2017). Meta-Analytic Bayesian Model For Differentiating Intestinal Tuberculosis from Crohn's Disease. Am J Gastroenterol.

[20] Jin T, Fei B, Zhang Y, Li KQ, Hao K, Zhang W, Fei BY (2017). The diagnostic value of polymerase chain reaction for Mycobacterium tuberculosis to distinguish intestinal tuberculosis from crohn's disease: a meta-analysis. Saudi J Gastroenterol.

[21] Lee JM, Lee KM (2016). Endoscopic diagnosis and differentiation of inflammatory bowel disease. Clin Endosc.

[22] Gan H, Mely M, Zhao J, Zhu L (2016). An analysis of the clinical, endoscopic, and pathologic features of intestinal tuberculosis. J Clin Gastroenterol.

[23] Jung Y, Hwangbo Y, Yoon SM, Koo HS, Shin HD, Shin JE, Moon HS, Kang SB, Lee JR, Huh KC (2016). Predictive factors for differentiating between Crohn's disease and intestinal tuberculosis in Koreans. Am J Gastroenterol.

[24] Zumla A, George A, Sharma V (2015). Herbert RH; baroness Masham of Ilton, Oxley a, et al. the WHO 2014 global tuberculosis report—further to go. Lancet Glob Health.

[25] MacLean E, Sulis G, Denkinger CM, Johnston JC, Pai M, Ahmad KF. Diagnostic accuracy of stool Xpert MTB/RIF for the detection of pulmonary tuberculosis in children: a systematic review and meta-analysis. J Clin Microbiol. 2019;57(6):e02057–18. 10.1128/JCM.02057-18 PMC6535592 30944200

[26] Bellam BL, Mandavdhare HS, Sharma K, Shukla S, Soni H, Kumar-MP (2019). Utility of tissue Xpert-Mtb/Rif for the diagnosis of intestinal tuberculosis in patients with ileocolonic ulcers. Ther Adv Infect Dis.

[27] Kumar S, Bopanna S, Kedia S, Mouli P, Dhingra R, Padhan R, Kohli M, Chaubey J, Sharma R, Das P, Dattagupta S, Makharia G, Sharma SK, Ahuja V (2017). Evaluation of Xpert MTB/RIF assay performance in the diagnosis of abdominal tuberculosis. Intest Res.

[28] Piersimoni C, Gherardi G, Gracciotti N, Pocognoli A (2019). Comparative evaluation of Xpert MTB/RIF and the new Xpert MTB/RIF ultra with respiratory and extra-pulmonary specimens for tuberculosis case detection in a low incidence setting. J Clin Tuberc Other Mycobact Dis.

[29] Chakravorty S, Simmons AM, Rowneki M, Parmar H, Cao Y, Ryan J (2017). The New Xpert MTB/RIF Ultra: Improving Detection of Mycobacterium tuberculosis and Resistance to Rifampin in an Assay Suitable for Point-of-Care Testing. mBio.

[30] Leung KS, Siu GK, Tam KK, Ho PL, Wong SS, Leung EK (2018). Diagnostic evaluation of an in-house developed single-tube, duplex, nested IS6110 real-time PCR assay for rapid pulmonary tuberculosis diagnosis. Tuberculosis (Edinb).

[31] Lim JH, Kim CK, Bae MH (2019). Evaluation of the performance of two real-time PCR assays for detecting Mycobacterium species. J Clin Lab Anal.

[32] Rahman MM, Rahim MR, Khaled A, Nasir TA, Nasrin F, Hasan MA (2017). Molecular detection and differentiation of Mycobacterium tuberculosis complex and non-tuberculous Mycobacterium in the clinical specimens by real time PCR. Mymensingh Med J.

[33] Patel B, Yagnik VD. Clinical and laboratory features of intestinal tuberculosis. Clin Exp Gastroenterol. 2018;11:97–103. 10.2147/CEG.S154235. 10.2147/CEG.S154235 PMC5856297 29559804

[34] Mehta V, Desai D, Abraham P, Gupta T, Rodrigues C, Joshi A, Deshpande R, Sawant P, Ingle M, Rathi P, Mandot A (2018). Do additional colonoscopic biopsies increase the yield of Mycobacterium tuberculosis culture in suspected ileo-colonic tuberculosis?. Indian J Gastroenterol.

[35] Diriba G, Kebede A, Yaregal Z, Getahun M, Tadesse M, Meaza A, et al. Performance of Mycobacterium growth Indicator tube BACTEC 960 with Lowenstein-Jensen method for diagnosis of Mycobacterium tuberculosis at Ethiopian National Tuberculosis Reference Laboratory, Addis Ababa. BMC Res Notes. 2017;10(1):181. 10.1186/s13104-017-2497-9. 10.1186/s13104-017-2497-9 PMC5424417 28486950

[36] Coppola M, Ottenhoff TH. Genome wide approaches discover novel Mycobacterium tuberculosis antigens as correlates of infection, disease, immunity and targets for vaccination. Semin Immunol. 2018;39:88–101. 10.1016/j.smim.2018.07.001. 10.1016/j.smim.2018.07.001 30327124

[37] Da Silva RJ, da Silva CR, Sardella IG, de Paulo Mulinari AC, Mafort TT, Santos AP, et al. IgA and IgG antibody detection of mycobacterial antigens in pleural fluid and serum from pleural tuberculous patients. BMC Immunol. 2019;20(1):36. 10.1186/s12865-019-0315-y. 10.1186/s12865-019-0315-y PMC6798396 31623558

[38] Meier NR, Jacobsen M, Ottenhoff THM, Ritz N. A systematic review on novel Mycobacterium tuberculosis antigens and their discriminatory potential for the diagnosis of latent and active tuberculosis. Front Immunol. 2018;9:2476. 10.3389/fimmu.2018.02476. 10.3389/fimmu.2018.02476 PMC6237970 30473692

[39] Ihama Y, Hokama A, Hibiya K. Diagnosis of intestinal tuberculosis using a monoclonal antibody to Mycobacterium tuberculosis. World J Gastroenterol. 2012;18(47):6974–80. 10.3748/wjg.v18.i47.6974. 10.3748/wjg.v18.i47.6974 PMC3531682 23322996

[40] Ince AT, Güneş P, Senateş E, Sezikli M, Tiftikçi A, Ovünç O. Can an immunohistochemistry method differentiate intestinal tuberculosis from Crohn's disease in biopsy specimens? Dig Dis Sci. 2011;56(4):1165–70. 10.1007/s10620-010-1399-7. 10.1007/s10620-010-1399-7 20824497

[41] Jørstad MD, Marijani M, Dyrhol-Riise AM, Sviland L, Mustafa T. MPT64 antigen detection test improves routine diagnosis of extrapulmonary tuberculosis in a low-resource setting: A study from the tertiary care hospital in Zanzibar. PLoS One. 2018;13(5):e0196723. 10.1371/journal.pone.0196723 PMC5942825 29742144

[42] Purohit MR, Sviland L, Wiker H, Mustafa T. Rapid and specific diagnosis of Extrapulmonary tuberculosis by Immunostaining of tissues and aspirates with anti-MPT64. Appl Immunohistochem Mol Morphol. 2017;25(4):282–8. 10.1097/PAI.0000000000000300. 10.1097/PAI.0000000000000300 26766121

[43] Sharma S, Sheoran A, Gupta KB, Yadav A, Varma-Basil M, Sreenivas V, et al. Quantitative detection of a cocktail of mycobacterial MPT64 and PstS1 in tuberculosis patients by real-time immuno-PCR. Future Microbiol. 2019;14(3):223–33. 10.2217/fmb-2018-0284. 10.2217/fmb-2018-0284 30663893

